# Gender Influence on White Matter Microstructure: A Tract-Based Spatial Statistics Analysis

**DOI:** 10.1371/journal.pone.0091109

**Published:** 2014-03-06

**Authors:** Richard A. Kanaan, Christopher Chaddock, Matthew Allin, Marco M. Picchioni, Eileen Daly, Sukhi S. Shergill, Philip K. McGuire

**Affiliations:** 1 University of Melbourne, Department of Psychiatry, Austin Health, Heidelberg, Victoria, Australia; 2 King’s College London, Institute of Psychiatry, Department of Psychosis Studies, London, United Kingdom; 3 St Andrew’s Academic Centre, King’s College London, Northampton, United Kingdom; 4 King’s College London, Institute of Psychiatry, Department of Developmental Psychiatry, London, United Kingdom; West China Hospital of Sichuan University, China

## Abstract

**Background:**

Sexual dimorphism in human brain structure is well recognised, but less is known about gender differences in white matter microstructure. We used diffusion tensor imaging to explore gender differences in fractional anisotropy (FA), an index of microstructural integrity. We previously found increased FA in the corpus callosum in women, and increased FA in the cerebellum and left superior longitudinal fasciculus (SLF) in men, using a whole-brain voxel-based analysis.

**Methods:**

A whole-brain tract-based spatial statistics analysis of 120 matched subjects from the previous analysis, and 134 new subjects (147 men and 107 women in total) using a 1.5T scanner, with division into tract-based regions of interest.

**Results:**

Men had higher FA in the superior cerebellar peduncles and women had higher FA in corpus callosum in both the first and second samples. The higher SLF FA in men was not found in either sample.

**Discussion:**

We confirmed our previous, controversial finding of increased FA in the corpus callosum in women, and increased cerebellar FA in men. The corpus callosum FA difference offers some explanation for the otherwise puzzling advantage in inter-callosal transfer time shown in women; the cerebellar FA difference may be associated with the developmental motor advantage shown in men.

## Introduction

It has long been recognised that the structure of the human brain differs between the sexes. Men have larger brains overall and in most regions, though women have a higher proportion of grey matter [1], [2]. There is some evidence that it is the white matter fraction that varies with sex, independently of brain size [3], but it is little studied in comparison with grey matter. Recent studies have begun to address this using diffusion-tensor magnetic resonance imaging (DTI).

DTI uses a magnetic resonance imaging (MRI) sequence sensitised to the diffusion of water, and by acquiring a measure of the diffusion in all directions inferences about the microstructure of white matter can be drawn [4]. The extent to which the diffusion follows the principal diffusion direction (the fractional anisotropy (FA)) can be informative about the cellular organization and myelination of white matter [5], changes that may be undetectable by volumetric MRI.

A number of studies [6]–[21] have used DTI to compare white matter microstructure between genders but these have tended to focus on particular regions of interest (ROIs), or have been limited by small samples, and have yielded conflicting results.

We recently published a paper [14] that set out to address some of these limitations, looking at white matter microstructure in 135 subjects using a whole-brain voxel-based analysis. We found women had higher FA in the corpus callosum (confirmed by ROI), whereas men had higher FA in the cerebellum and left anterior superior longitudinal fasciculus (SLF). These results were themselves open to question, however, as the direction of the corpus callosum difference we found conflicted with much of the rest of field, and the method we used poorly localised clusters of difference (in the cerebellum and SLF) and was inherently vulnerable to partial volume effect (especially in the SLF cluster), so that we were concerned that the SLF difference in particular may have been spurious. We therefore proposed to confirm the results using a method (tract-based spatial statistics, or TBSS [22]) that suffers reduced partial volume effect as it uses the maximal value for each tract at each location, and which we adapted to give more specific localisation, hypothesizing that all of our results would be confirmed except that from the SLF. We then proposed to replicate these results in a second, independent sample, using the same method.

## Methods

### Ethics Statement

All subjects gave written, informed consent after the study was explained to them, and the study was approved by the Institute of Psychiatry, King’s College London, Research Ethics Committee.

### Subjects

We took the scans of healthy volunteers from our previous VBA (voxel-based analysis) [14] and added scans from a larger set of healthy volunteers. We excluded all who were not right handed, and closely matched the remaining gender groups on key demographics. After excluding two male subjects whose scans did not successfully undergo the image processing procedure, there remained 254 subjects (147 men, 107 women), 120 from the original study and 134 new subjects. The demographics of the original and new samples were significantly different (by t-test or Chi-squared) - see table 1. The gender groups were matched for handedness (all dextral), age (men 32.4+/−12.6, women 32.7+/−13.5; range 18–63; Mann Whitney p = 0.90) and IQ (men 110.1+/−11.0, women 110.7+/−12.2; t-test p = 0.74). The gender groups also matched on ethnicity (p = 0.32) and parental social class (p = 0.73), though there were extensive missing values for these measures (119 men/56 women were missing class data, and 84 men/47 women were missing ethnicity data).

**Table 1:** Demographics of the original and new samples.

	Original Sample (n = 120)	New Sample (n = 134)	
Mean Age (SD)	24.7 (6.5)	39.6 (13.2)	p<0.001
Gender (male/female)	81/39	66/68	p = 0.003
Mean IQ (SD)	108.9 (10.7)	111.9 (12.2)	p = 0.06
Mean Years of Education (SD)	15.1 (2.6)	15.2 (2.7)	p = 0.9
Ethnicity (C/AC/A/O)	24/2/1/6	83/4/1/2	p = 0.01
Social Class of Parents (I/II/III/IV/V)	1/2/3/1/0	15/24/32/3/5	p = 0.7

SD: standard deviation; C: Caucasian; AC: African-Caribbean; A: Asian; O: Other.

### Image Acquisition and Pre-processing

Diffusion-weighted imaging data were acquired using a GE Signa 1.5 Tesla LX MRI system (General Electric, Milwaukee, Wisconsin, USA) with a standard birdcage quadrature head coil, using an echo planar imaging sequence peripherally gated to the cardiac cycle and optimised for the acquisition of white matter diffusion tensor MRI. Seven non-diffusion-weighted images (*b* = 0) were acquired, along with 64 images with diffusion gradients (*b* = 1300 s/mm2) uniformly distributed in space [23] at each of 60 slices. The TR was 15 cardiac R-R intervals with a TE of 107 ms. Whole-head acquisition gave isotropic (2.5 mm^3^) voxels, reconstructed to a 1.875×1.875 mm in-plane pixel size. Following a mutual-information image correction (see [24]), in-house software was used to remove non-brain tissue, determine the diffusion tensor and calculate the fractional anisotropy (FA) in each remaining voxel [25].

### Tract-based Spatial Statistics

Between-group FA comparisons were conducted using TBSS version 1.2 [22]. FA images from all participants were aligned to the Johns Hopkins University – International Consortium of Brain Mapping DTI-81 white matter atlas (JHU DTI atlas) [26] using FMRIB’s non-linear image registration tool (FNIRT) in FSL (http://fsl.fmrib.ox.ac.uk/fsl/fslwiki/). The mean of the voxel-wise FA images was ‘skeletonised’ (to generate a study-specific mean FA ‘skeleton’ representing the centres of tracts common to all participants) and thresholded for white matter (FA >0.3). The aligned maps were then projected onto the mean white matter skeleton, and then subdivided according to the 48 regions of the JHU DTI atlas, with FA averaged per region per-subject, and these regional means compared between groups using IBM SPSS v20 *(* www.ibm.com/software/analytics/spss). The regions compared were the three main divisions of the corpus callosum, the three cerebellar tracts and the superior longitudinal fasciculus, corresponding to the areas of difference reported in our previous study. The first comparison was between the male and female subjects from our previous study, followed by the new subjects, and finally by the combined sample. As FA data is not normally distributed (and was not in our study - p<0.05, Shapiro-Wilk) the principal comparisons used were Mann-Whitney U tests. Effect sizes were calculated using the Glass rank biserial correlation coefficient.

## Results

Comparing the projected skeletons by tract-ROI-averages from the original sample gave the results in table 2. This confirmed and localised the results of our previous analysis for the corpus callosum and cerebellum: more specifically, we found higher FA in women in the genu of the corpus callosum; we found higher FA in men in the bilateral superior cerebellar peduncles, but not the rest of the cerebellum. The superior longitudinal fasciculus did not differ between sexes, as hypothesized. Though our previous analysis reported higher female FA in the genu and body of the corpus callosum, the body and splenium were, on this analysis, not significant, though a trend was detected in the splenium.

**Table 2:** Tract-averaged voxel-wise FA compared between genders, original sample.

Tract Name	Median FA Male (IQR)	Median FA Female (IQR)	Effect Size	p-value
**Corpus Callosum**				
Genu	0.710 (.04)	0.734 (.03)	0.41	.000**
Body	0.688 (.04)	0.697 (.03)	0.18	.102
Splenium	0.769 (.03)	0.774 (.03)	0.20	.074
**Superior Longitudinal Fasciculus**				
Left	0.523 (.03)	0.526 (.02)	0.11	.323
Right	0.521 (.03)	0.526 (.03)	0.07	.521
**Cerebellar Peduncles**				
Superior Left	0.617 (.06)	0.599 (.04)	−0.29	.011*
Superior Right	0.615 (.05)	0.602 (.04)	−0.25	.024*
Middle	0.560 (.03)	0.551 (.02)	−0.20	.079
Inferior Left	0.534 (.04)	0.538 (.03)	0.03	.807
Inferior Right	0.549 (.04)	0.547 (.02)	0.09	.405

*p<0.05;

**p<0.005. IQR: interquartile range.

Comparing the 134 new subjects gave the results in table 3, which closely, but not precisely match those in table 2: FA was again higher in the corpus callosum (but this time genu and splenium); FA was again higher in the superior cerebellar peduncles in men. While FA in the left SLF was significantly different, as in the prior VBA analysis, this does not represent a confirmation as the direction of difference was reversed, with FA now higher in women.

**Table 3:** Tract-averaged voxel-wise FA compared between genders, new sample.

Tract Name	Median FA Male (IQR)	Median FA Female (IQR)	Effect Size	p-value
**Corpus Callosum**				
Genu	0.709 (.05)	0.719 (.04)	0.20	.044*
Body	0.687 (.05)	0.690 (.05)	0.15	.138
Splenium	0.765 (.03)	0.772 (.02)	0.24	.019*
**Superior Longitudinal Fasciculus**				
Left	0.513 (.04)	0.524 (.04)	0.23	.019*
Right	0.514 (.03)	0.521 (.04)	0.16	.101
**Cerebellar Peduncles**				
Superior Left	0.641 (.05)	0.619 (.05)	−0.29	.003**
Superior Right	0.633 (.05)	0.617 (.04)	−0.26	.010*
Middle	0.552 (.03)	0.556 (.02)	0.02	.873
Inferior Left	0.528 (.05)	0.530 (.03)	−0.01	.915
Inferior Right	0.535 (.05)	0.542 (.03)	0.03	.762

*p<0.05;

**p<0.005. IQR: interquartile range.

Combining the samples gave the results in table 4, which unsurprisingly again found FA higher in the corpus callosum (genu and splenium); FA was again higher in the superior cerebellar peduncles in men. The left SLF was significantly higher in women.

**Table 4:** Tract-averaged voxel-wise FA compared between genders, combined sample.

Tract Name	Median FA Male (IQR)	Median FA Female (IQR)	Effect Size	p-value
**Corpus Callosum**				
Genu	0.710 (.04)	0.726 (.04)	0.27	.000**
Body	0.688 (.04)	0.693 (.04)	0.13	.082
Splenium	0.768 (.03)	0.773 (.03)	0.21	.005*
**Superior Longitudinal Fasciculus**				
Left	0.520 (.04)	0.525 (.03)	0.15	.042*
Right	0.518 (.03)	0.523 (.04)	0.10	.186
**Cerebellar Peduncles**				
Superior Left	0.631 (.05)	0.612 (.04)	−0.22	.002**
Superior Right	0.620 (.05)	0.611 (.05)	−0.20	.007*
Middle	0.558 (.03)	0.554 (.02)	−0.09	.239
Inferior Left	0.533 (.04)	0.533 (.03)	−0.03	.677
Inferior Right	0.543 (.04)	0.545 (.03)	0.02	.767

*p<0.05;

**p<0.005. IQR: interquartile range.

Though our scans were of adults, and were closely matched for age, given the theoretical effects of differential white matter maturation between the sexes an interaction with age remains possible. We therefore individually power-transformed the variables of interest from the combined sample to normal distributions, and then entered them in an analysis of variance with age and gender as independent variables: in no case was there a significant age × gender interaction.

Though the hypotheses under investigation were in respect of the tracts identified by the previous VBA analysis, the analysis method we employed here also generated results for the other tracts, and we have included these in table 5 for interest. The significance of these differences, which involve a large number (48) of comparisons, depends critically on which multiple-comparison correction method is used. Using stringent Bonferroni correction, or Hochberg’s improved Bonferroni [27], only the genu, bilateral posterior thalamic radiations and the right ILF differences remain significant. Correcting using the more powerful [28] but more lenient False Discovery Rate [29], yielded significant differences in, additionally, the splenium, left cingulum/hippocampus, the right corticospinal tract, the bilateral retrolenticular internal capsule, bilateral superior cerebellar peduncles, left ILF and right superior fronto-occipital fasciculus (FOF). Following Cohen’s guidelines for a correlation coefficient, all the significant effect sizes were small (<0.3) but non-trivial (>0.1), except for the genu in the first analysis, which was of medium size (<0.5).

**Table 5:** Tract-averaged voxel-wise FA compared between genders, combined sample, other regions of interest.

Tract Name	Median FA Male (IQR)	Median FA Female (IQR)	Effect Size	p-value
Anterior Corona Radiata L	0.484 (.04)	0.481 (.04)	−0.01	.839
Anterior Corona Radiata R	0.489 (.04)	0.490 (.04)	−0.04	.600
Anterior Limb Internal Capsule L	0.570 (.03)	0.574 (.03)	0.09	.224
Anterior Limb Internal Capsule R	0.590 (.03)	0.592 (.03)	0.02	.790
Cerebral Peduncle L	0.685 (.03)	0.683 (.03)	−0.01	.891
Cerebral Peduncle R	0.699 (.03)	0.696 (.04)	−0.09	.247
Cingulum/Hippocampus L	0.533 (.05)	0.518 (.05)	−0.21	.005*
Cingulum/Hippocampus R	0.533 (.06)	0.526 (.05)	−0.09	.202
Cingulum L	0.615 (.05)	0.615 (.05)	−0.06	.396
Cingulum R	0.581 (.05)	0.583 (.04)	0.01	.844
Corticospinal tract L	0.568 (.04)	0.559 (.04)	−0.12	.090
Corticospinal tract R	0.557 (.04)	0.547 (.04)	−0.21	.004**
External Capsule L	0.466 (.03)	0.467 (.02)	0.00	.991
External Capsule R	0.464 (.03)	0.464 (.03)	0.00	.961
Fornix	0.479 (.07)	0.490 (.09)	0.08	.307
Fornix/stria terminalis L	0.550 (.05)	0.558 (.04)	0.15	.044*
Fornix/stria terminalis R	0.552 (.04)	0.550 (.04)	0.03	.672
Medial Lemniscus L	0.570 (.04)	0.569 (.04)	−0.10	.160
Medial Lemniscus R	0.587 (.04)	0.577 (.04)	−0.18	.016*
Pontine crossing tracts	0.495 (.03)	0.494 (.04)	−0.03	.665
Posterior Corona Radiata L	0.496 (.03)	0.501 (.03)	0.10	.193
Posterior Corona Radiata R	0.509 (.03)	0.514 (.04)	0.10	.154
Posterior Limb Internal Capsule L	0.670 (.03)	0.671 (.03)	0.04	.588
Posterior Limb Internal Capsule R	0.664 (.03)	0.669 (.03)	0.06	.427
Posterior Thalamic Radiations L	0.608 (.04)	0.622 (.04)	0.26	.000**
Posterior Thalamic Radiations R	0.605 (.04)	0.621 (.03)	0.32	.000**
Retrolenticular Internal Capsule L	0.598 (.03)	0.610 (.04)	0.22	.003**
Retrolenticular Internal Capsule R	0.583 (.03)	0.591 (.03)	0.20	.007*
Sagittal Stratum/ILF L	0.540 (.03)	0.555 (.04)	0.23	.002**
Sagittal Stratum/ILF R	0.546 (.04)	0.561 (.04)	0.25	.001**
Superior Corona Radiata L	0.500 (.03)	0.504 (.03)	0.07	.334
Superior Corona Radiata R	0.491 (.03)	0.497 (.03)	0.03	.645
Superior FOF L	0.479 (.04)	0.483 (.04)	0.05	.465
Superior FOF R	0.480 (.04)	0.495 (.03)	0.20	.006*
Tapetum L	0.593 (.07)	0.602 (.09)	0.11	.141
Tapetum R	0.549 (.09)	0.560 (.08)	0.16	.030*
Uncinate L	0.483 (.07)	0.481 (.05)	0.01	.874
Uncinate R	0.527 (.06)	0.515 (.06)	−0.13	.070

*p<0.05;

**p<0.005. IQR: interquartile range; L = left; R = right; ILF = Inferior Longitudinal Fasciculus; FOF = Fronto-Occipital Fasciculus.

## Discussion

We confirmed, as hypothesized, higher FA in the corpus callosum; we confirmed higher FA in the superior cerebellar peduncles but not in the left anterior SLF in men. These confirmations in our first sample using a different method, as well as in a second, independent sample of equal size, give us greater confidence that the results may prove true, at least for samples like ours. Though our two samples markedly differed (table 1) both were largely Caucasian, relatively well-educated, right-handed samples, and interactions with handedness in particular cannot be excluded. And more generally, in a study such as this where the effect sizes are small, there are any number of unmeasured variables (such as oral contraception [30]) that might potentially confound the results.

The meaning of these FA differences in healthy subjects is not straightforward. FA is one of many diffusion indices, and has a number of determinants in white matter, including tissue architecture, myelination, fiber diameter and density, such that it is not normally possible to anatomically interpret an FA difference without at least an additional myelin measurement such as T2-relaxometry [31]. However, when FA is found to be influenced by disease, this is almost always to reduce FA [32], and in the healthy, higher FA has been associated with advantage, such as a correlation with increased conduction speed [33], [34] or reduced reaction time [35]. So, it is plausible to consider higher FA in these study as reflecting a gender-differentiated functional adaptation or specialization.

One important potential confound in this is structure size, as smaller structures predispose to partial volume artefacts [31], and female brains are typically smaller, with smaller white matter, as noted in the introduction. In our results, however, most of the differences were in favour of higher FA in women, such that this would tend to be conservative, and we chose a method of analysis that should minimize the partial volume effect, though it cannot exclude it entirely (as you might, for example, with a concurrent volumetric analysis). Another possible confounder is age, even in a matched sample, as the differential developmental trajectory of white matter by gender is clearly described, with girls’ white matter generally maturing earlier in adolescence than boys, particularly in frontal areas [36]. Ours was an adult sample so that both groups should be fully mature, but any lingering maturational delay in men might be expected to result in higher FA in female brains, particularly frontally. However, there was no evidence of an interaction between age and gender in this sample when this was examined.

Our finding of higher FA in the corpus callosum in females is a potentially important contribution to the rich literature on gender differences in this tract. There have been a number of previous, typically small, studies of FA, but the results of these stand in stark conflict with each other, with some reporting higher FA in men [7], [8], [10], [15], [37], some finding no difference [12], [16], [38] and some finding increases in women [6], [39]. Our previous study sided with the minority finding of increased FA in women [14], a finding that we felt made sense of the enhanced callosal connectivity in women shown using graph theory [40], [41] and interhemispheric transfer time [42]. As interhemispheric transfer time generally *decreases* with area [43], [44], females should be expected to have greater (i.e. slower) transfer times. The finding of higher FA in women in the corpus callosum, with the possible exception of the splenium, offers a clue to a greater efficiency that might explain why this is not the case.

The cerebellum is less studied than the corpus callosum in regard to sexual dimorphism. Most studies report larger cerebellar volumes in men [1], [45]–[47], though some have found this to be a function of brain volume [48], [49], with one study finding women to have a larger cerebellar volume relative to total brain volume [50]. We are aware of only three smaller studies that have considered gender differences in cerebellar microstructure, none reporting differences [19], [20], [37]. Notably, the differences we found were specific to the superior cerebellar peduncles, a relatively small tract (by volume) when compared with the rest of cerebellar white matter, which may partially explain this discrepancy. Gender differences in motor function are among the more robust [51], and associate with gender volume differences in children [52]. It is tempting to see an association between these and the FA differences, though unlike the corpus callosum this would not be a reciprocal relationship.

The ‘loss’ of the previously reported superior longitudinal fasciculus FA increase in men was hypothesized, as we suspected a partial volume effect in the earlier study. The finding had not been noted in any previous research, and we could not, in that study, find a convincing explanation for its presence [14]. Though not under hypothesis, we also found higher FA in the bilateral posterior thalamic radiations and retrolenticular internal capsules in women. The posterior thalamic radiations are formed by fibers passing through the retrolenticular internal capsule (part of the posterior limb), including thalamic pathways, optic radiations, long corticofugal pathways and cortico-cortical association tracts such as the inferior longitudinal fasciculus. Given that the sagittal stratum/inferior longitudinal fasciculus was also bilaterally elevated in females, we would be inclined to interpret these as parts of a single tract difference, but this would await confirmation from a targeted study.
